# Decreased renal function among children born to women with obstructed labour in Eastern Uganda: a cohort study

**DOI:** 10.21203/rs.3.rs-3121633/v1

**Published:** 2023-07-12

**Authors:** David Mukunya, Faith Oguttu, Brendah Nambozo, Ritah Nantale, Tonny Brian Makoko, Agnes Napyo, Josephine Tumuhamye, Solomon Wani, Prossy Auma, Ketty Atim, Dedan Okello, Joan Wamulugwa, Lawrence Ssegawa, Julius Wandabwa, Sarah Kiguli, Martin Chebet, Milton W Musaba, Doreck Nahurira

**Affiliations:** Busitema University; Busitema University; Busitema University; Busitema University; Busitema University; Busitema University; Busitema University Centre of Excellency for Maternal Reproductive and Child Health; Busitema University; Mbale Regional Referral Hospital; Mbale Regional Referral Hospital; Mbale Regional Referral Hospital; Mbale Regional Referral Hospital; Sanyu Africa Research Institute; Busitema University; Makerere University Hospital, Makerere University Kampala; Busitema University; Busitema University; Busitema University

**Keywords:** Renal function, children, obstructed labour, eGFR, Uganda

## Abstract

**Background:**

Over two million children and adolescents suffer from chronic kidney disease globally. Early childhood insults such as birth asphyxia could be risk factors for development of chronic kidney disease in infancy. Our study aimed to assess renal function among children aged two to four years, born to women with obstructed labour.

**Methods:**

We followed up 144 children aged two to four years, born to women with obstructed labor at Mbale regional referral hospital in Eastern Uganda. We used estimated glomerular filtration rate (eGFR) by the Schwartz formula to calculate eGFR (0.413*height)/ serum creatinine as a measure of renal function. eGFR less than 90 ml/min/1.73m^2^ was classified as decreased renal function.

**Results:**

The mean age of the children was 2.8 years, standard deviation (SD) of 0.4 years. Majority of the children were male (96/144: 66.7%). The mean umbilical lactate level at birth among the study participants was 8.9 mmol/L with a standard deviation (SD) of 5.0. eGFR values ranged from 55 to 163ml/min/1.72m^2^, mean 85.8 ± SD 15.9. One third (31.3%) 45/144 had normal eGFR (> 90 ml/Min/1.72m^2^), two thirds (67.4%) 97/144 had a mild decrease of eGFR (60–89 ml/Min/1.72m^2^), and only 2/144 (1.4%) had a moderate decrease of eGFR. Overall incidence of reduced eGFR was 68.8% (99/144).

**Conclusion:**

We observed a high incidence of impaired renal function among children born to women with obstructed labour. We recommend routine follow up of children born to women with obstructed labour and add our voices to those calling for improved intra-partum and peripartum care.

## Introduction

Chronic kidney disease is one of the most common pediatric noncommunicable diseases, affecting over 2 million children and adolescents globally [1]. Chronic kidney disease results in 1.2 million deaths annually and 35.8 million disability-adjusted life-years [2]. Low- and middle-income countries contribute 70% of the global burden of chronic kidney disease. Sub-Saharan Africa has an increasing burden of noncommunicable diseases. By 2030, more than two thirds of the global end-stage renal disease is expected to be in low-income countries[3]. The prevalence of chronic kidney disease is estimated at 14% in sub-Saharan Africa, and 15% in Uganda [4]. There is urgent need to identify risk factors for chronic kidney disease so that timely and effective interventions are instituted.

The life course epidemiology approaches suggest that insults in the peripartum period could result in non-communicable diseases such as chronic kidney disease in later life [5]. One potential insult in the peripartum period is obstructed labour. Obstructed labour complicates 22% of pregnancies [6], and is involved in 50% of perinatal deaths [7] and 9% of maternal deaths [6] in Uganda and some parts of Africa. Unrelieved obstruction is associated with fetal asphyxia, which results in shunting of blood from the kidneys to maintain cerebral, cardiac, and adrenal perfusion [8]. Under-perfusion of the kidney is followed by necrotic and apoptotic cell injury to the cortical and tubular renal parenchymal cells hence acute kidney injury. Recovery from acute kidney injury by maladaptive repair results in alteration of the kidney structure hence irreversible damage [9–11].

Recent advances in chronic kidney disease highlight the role of neonatal insults in its pathogenesis [5]. Approximately 27–67% of survivors of neonatal acute kidney injury present with impaired renal function at two to three years [9, 12, 13]. However, it is not clear whether the kidney damage following obstructed labour persists into infancy. Early identification of renal dysfunction could result in timely interventions which could be life and organ saving. Therefore, we aimed to determine renal function among children aged 2 to 4 years, born to women with obstructed labour in Mbale regional referral hospital.

## Methods

### Aim

To assess renal function among children aged two to four years, born to women with obstructed labour

### Study design

We conducted a prospective cohort study among children aged two to four years, born to women with obstructed labour. These women had participated in a double blind, randomized controlled trial to establish the effect of sodium bicarbonate on maternal and perinatal outcomes among women with obstructed labour in Mbale regional referral hospital between July 2018 and September 2019 [14], **Trial registration number;** PACTR201805003364421[19].

### Study setting

The study was conducted in Mbale regional referral and teaching hospital between October 2021 to April 2022. Mbale regional referral hospital is a public hospital that serves 13 districts (with about 4 million people in the region). It is the main referral center for four district hospitals and 10 health sub-districts in and around Elgon sub-region. The hospital is staffed by 4 paediatricians, runs 4 special clinics: diabetic clinic, paediatric psychiatry clinic, neonatal clinic and a chronic care clinic. The chronic care clinic runs every Wednesday for children with sickle cell disease, kidney disease, heart disease. Annually, about 5% (600) of the women that deliver in Mbale regional referral hospital are diagnosed with obstructed labour [15].

### Study participants

Our participants were children aged between two and four years, born to women with obstructed labour. These women participated in a clinical trial between July 2018 and September 2019 to determine the effect of a sodium bicarbonate infusion on blood lactate, maternal and perinatal outcomes among women with obstructed labour in Mbale regional referral hospital [14].

### Inclusion criteria

Children aged 2 to 4 years born to women with obstructed labour in the PACTR201805003364421 trial [14], alive and willing to come to Mbale regional referral hospital for follow up.

### Exclusion criteria

We excluded children whose care takers declined to have a a blood sample collected.

### Outcome variables

Our outcome of interest was renal function defined as estimated glomerular filtration rate (eGFR) calculated by the Schwartz formula for eGFR ((0.413*height)/creatinine) using a Stata ado file egfr, f(schwartz) cr(SCreatinine) s height(child_avg_ht)[16].

Normal eGFR was defined as greater than or equal to 90 ml/min/1.73m^2^. Reduced eGFR was defined as eGFR less than 90ml/min/1.73m^2^. Mild decrease of eGFR was defined as eGFR 60 to 89 ml/min/1.73m^2^ while moderate decrease of eGFR was defined as eGFR less than 60 ml/min/1.73m^2^.

### Independent variables:

We measured socio-demographic characteristics (maternal age, wealth index, child’s age in months, and sex of the child), anthropometric measurements, umbilical lactate at birth, stunting, exclusive breast feeding, and wealth index. We ascertained the presence of alternative causes of renal dysfunction in this population such as diabetes by conducting a random blood glucose measurement and HIV/AIDS using an ELISA strip. We also collected urine samples from the children for urinalysis.

### Data collection

#### Clinical assessment

Once informed consent was provided and eligibility confirmed, the following information and specimens were obtained:

1. Clinical history included assessment of the mother’s obstetric history, child’s feeding history, pastmedical history.
2. Physical examination included assessment of the child’s neurological function, and anthropometric measurements (height, weight, mid upper arm circumference and head circumference) according to WHO/UNICEF guidelines and as outlined in the manual of procedures. Routine testing for HIV was performed. All children known to have HIV underwent staging by WHO/UNICEF guidelines.
3. Collection of urine sample. After consent, the caretaker was given a urine bottle and instructed on how to collect a midstream sample; whenever the child wants to urinate.
4. Under aseptic conditions, we obtained approximately 3mls of blood using a single-use 5ml syringe by venipuncture. The cubital fossa was first cleaned with alcohol swabs and dried with cotton prior to blood collection. Blood was then processed for Serum Creatinine and Urea, Random Blood Sugar and HIV testing. HIV tests were carried out according to Ministry of Health testing procedures using two rapid enzyme linked immunoassays (Determine HIV-1/2, Abbott Labouratories, USA; Uni-Gold, Trinity Biotech PLC, Ireland) and a third rapid test (HIV 1/2 STAT-PAK, Chembio diagnostics, USA) if results are discordant. A drop of blood was put on the Random Blood Sugar Kit and the HIV ELISA KIT. Random blood glucose was measured in mmol/L using On Call^®^ Plus glucometer (ACON Laboratories, Inc., 10125 Mesa Road, San Diego, California, USA), a point-of-care test. Blood for Serum creatinine was collected in a red top vacutainer and analyzed using a COBAS-INTEGRA 400 machine and at the lancet laboratories in Mbale (South African National Accreditation System Reg. No: 1996/006959/07).

### Sample size and sampling

The sample size was dependent on the size of the parent study whose aim was to determine the prevalence of neurodevelopmental delay in this cohort [17]. A total of 144 participants had a serum creatinine measurement. This sample size resulted in an absolute precision of 2.3–8.2%, i.e., the difference between the point estimate and the 95% confidence interval (CI) for prevalence values ranging from 2–50%, a precision we deemed adequate.

### Statistical analysis

Data were analyzed using Stata version 14.0 (Stata Corp; College Station, TX, USA). Continuous variables were summarized into means, median, and standard deviation. Categorical variables were summarized into proportions. Reduced eGFR was defined as eGFR less 90ml/min/1.72m^2^

Bivariable and multivariable analyses were explored using generalized linear model for the Poisson family with a log link to obtain prevalence ratios to assess the strength of associations between independent variables and reduced eGFR.

## Results

### Study profile

**Figure 1:**
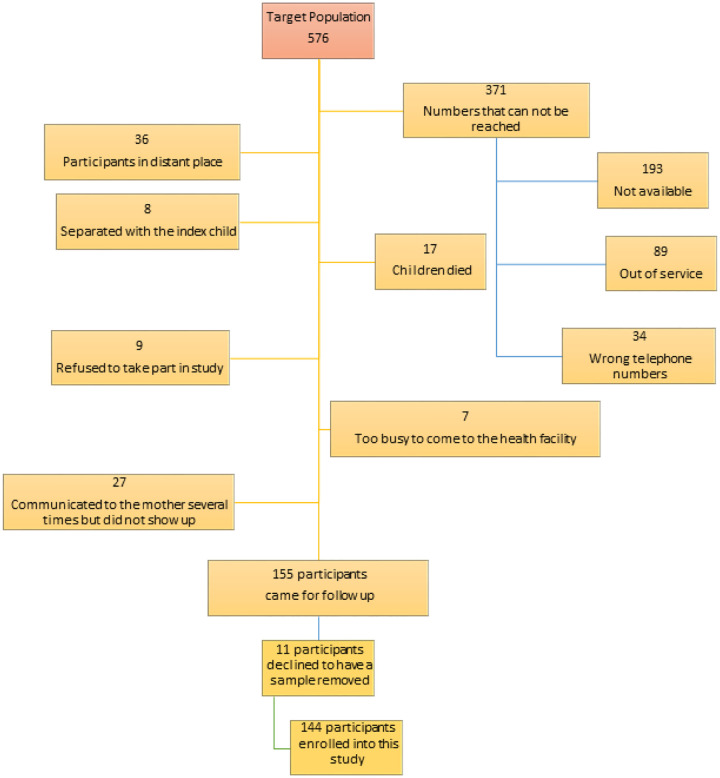
Study profile

In the clincial trial, we enrolled 576 children. Out of these, 155 came for follow up [17] and 144 consented to have blood samples picked for analysis. Details of patient flow and reasons for failure to come for follow up are indicated in Fig. 1.

### Participant characteristics

We enrolled 144 children with a mean age of 34.0 months standard deviation (SD) of 4.6 months. Most of the participants were male 96/144 (66.7%), only 23/144 (16%) were admitted during the neonatal period, 58/144 (40.3%) were exclusively breastfed and 7/144 (4.9%) were stunted. The mean umbilical lactate level at birth among the study participants was 8.9 mmol/L with a standard deviation (SD) of 5.0. Table 1. Summarizes the socio demographic characteristics, nutrition and past medical history of children born to women with obstructed labour in Eastern Uganda.

### Reduced eGFR among children born to women with obstructed labour in Eastern Uganda.

Overall incidence of reduced eGFR was 68.8% (99/144). Of the 144 children that were followed up 45(31.3%) had normal eGFR (> 90 ml/min/1.73m^2^), 97(67.4%) had mild decrease of eGFR (60–89 ml/min/1.73m^2^). Only 2(1.4%) had a moderate decrease of eGFR. Reduced eGFR was defined as less than 90ml/min/1.73m^2^. eGFR ranged from 55–163ml/min/1.73m^2^, mean 85.77 ± SD 15.9, median 83.8.

At both bivariable and multivariable analysis, none of the factors were found to be associated with reduced eGFR at two to four years. Table 2 summarizes factors associated with reduced eGFR.

## Discussion

This study found a high incidence of reduced estimated glomerular filtration rate (eGFR) 99/144 (68.8%). Of the 99 children with reduced eGFR, 97 had mild decrease of eGFR (60–89 ml/min/1.73m^2^) and only 2 had a moderate decrease of eGFR. Despite the high incidence, none of the factors was associated with decreased renal function among children born to women with obstructed labour.

The incidence of decreased renal function was high in this study. During obstructed labour, the fetus is asphyxiated which results in shunting of blood from the kidneys to maintain cerebral, cardiac, and adrenal perfusion [8]. The under-perfusion of the kidney is followed by necrotic and apoptotic cell injury to the cortical and tubular renal parenchymal cells due to anaerobic and reperfusion injury hence acute kidney injury at birth. Recovery from acute kidney injury by maladaptive repair results in alteration of the kidney structure hence irreversible damage [9–11]. Our findings are consistent with a study done in British Columbia among 126 children less than one year admitted with acute kidney injury. The surviving children were assessed for risk of chronic kidney disease at 1 year, 2 years or 3 years and 60% had mildly decreased eGFR of 60–90 mL/min/1.73m^2^ [13].

Our incidence was much higher as compared to a retrospective study in the USA which followed up 80 children with nephrotoxic acute kidney injury secondary to aminoglycosides which found that only 23% had an eGFR < 90 mL/min/1.73 m^2^. This can be explained by the fact that this study involved much older children without history of birth asphyxia [18].

Our study found no association between umbilical arterial lactate and decreased renal function. The median umbilical arterial lactate in our study was 7.2 mmol/L which is way above the normal (≤ 4.8 mmol/L). As such, almost all children were exposed to a severe form of birth asphyxia and unit increases in umbilical arterial lactate could not make any more difference. Other studies that used Apgar score and grade of hypoxic-ischaemic encephalopathy as markers of degree of asphyxia found a correlation between severity of asphyxia and renal impairment at 96 hours of life [19] and 6 months of age [20]. We believe umbilicate arterial lactate is a more accurate measure of birth asphyxia.

### Strength and limitations

This study is the first of its kind to assess the kidney function among children born to mothers with obstructed labour. A major limitation of our study is the high loss to follow up, we speculate that the incidence of renal dysfunction may have been higher or lower with a bigger sample size. We believed that the presence of mildly decreased eGFR (60–90 mL/min/1.73 m^2^) was a valid estimate of renal function. There is some uncertainty regarding the relevance of the Schwartz formula which has been shown to overestimate true GFR by as much as 25%−30%. Furthermore, our study did not collect data on urine creatinine, so we were unable to determine albumin to creatinine ratio which is a good marker of kidney function.

## Conclusion

We found a high incidence of decreased renal function among children born to mothers with obstructed labour. These children could be at risk of chronic kidney disease. We recommend follow up and screening for renal impairment among children born to mothers with obstructed labour. Finally, we add our voices to those calling for improved intra-partum and peripartum care.

## Figures and Tables

**Table 1:** Socio demographic, anthropometric, and clinical characteristics of children born to women with obstructed labour in Eastern Uganda

	Normal eGFR ≥ 90 (mL/min/1.73 m^2^) N = 45	Reduced eGFR < 90 (mL/min/1.73 m^2^) N = 99	Total N = 144
**Age of the child**			
Less than 3 years	26(57.8)	66(66.7)	92(63.9)
3 years and above	19(42.2)	33(33.3)	52(36.1)
**Sex**			
Male	26(57.8)	70(70.7)	96(66.7)
Female	19(42.2)	29(29.3)	48(33.3)
**Mother’s educational level**			
Primary	17(37.8)	25(25.3)	42(29.2)
Secondary	17(37.8)	45(45.5)	62(43.1)
Tertiary	11(24.4)	29(29.3)	40(27.8)
**Wealth index**			
Q1. Poorest	9(20)	20(20.2)	29(20.1)
Q2	10(22.2)	20(20.2)	30(20.8)
Q3	9(20)	21(21.2)	30(20.8)
Q4	10(22.2)	18(18.2)	28(19.4)
Q5. Richest	7(15.6)	20(20.2)	27(18.8)
**Child’s birth order**			
First born	19(42.2)	50(50.5)	69(47.9)
2–4	16(35.6)	37(37.4)	53(36.8)
5–12	10(22.2)	12(12.1)	22(15.3)
**Umbilical arterial lactate (mmol/L)**			
<4.8	10(23.3)	21(21.2)	31(21.8)
4.8–10	19(44.2)	46(46.5)	65(45.8)
>10	14(32.6)	32(32.3)	46(32.4)
**Was the child ever admitted to Special Care Unit?**			
No	36(80)	85(85.9)	121(84)
Yes	9(20)	14(14.1)	23(16)
**Has your baby been admitted for any illness in the last one year?**			
No	34(75.6)	86(86.9)	120(83.3)
Yes	11(24.4)	13(13.1)	24(16.7)
**Oxygen after birth**			
No	39(86.7)	91(91.9)	130(90.3)
Yes	6(13.3)	8(8.1)	14(9.7)
**Neonatal fever**			
No	34(75.6)	90(90.9)	124(86.1)
Yes	11(24.4)	9(9.1)	20(13.9)
**Hospital admission for severe malaria in last one year**			
No	35(77.8)	88(88.9)	123(85.4)
Yes	10(22.2)	11(11.1)	21(14.6)
**HIV status**			
Negative	38(84.4)	91(91.9)	129(89.6)
Positive	7(15.6)	8(8.1)	15(10.4)
**Early breastfeeding initiation**			
No	24(53.3)	63(63.6)	87(60.4)
Yes	21(46.7)	36(36.4)	57(39.6)
**Food diversity**			
No	3(6.7)	15(15.2)	18(12.5)
Yes	42(93.3)	84(84.8)	126(87.5)
**Stunting**			
No	37(82.2)	78(78.8)	115(79.9)
Yes	8(17.8)	21(21.2)	29(20.1)
**Wasting**			
No	44(97.8)	93(93.9)	137(95.1)
Yes	1(2.2)	6(6.1)	7(4.9)
**Minimum meal frequency**			
< 4 meals a day	12(26.7)	33(33.3)	45(31.3)
4 and above meals	33(73.3)	66(66.7)	99(68.8)
**Exclusive breastfeeding**			
No	23(51.1)	63(63.6)	86(59.7)
Yes	22(48.9)	36(36.4)	58(40.3)
**Blood pressure**			
Normal blood pressure	36 (80%)	69 (70%)	105(73)
Elevated blood pressure	9 (20%)	30 (30%)	39(27)

**Table 2:** Factors associated with reduced eGFR among children born to women with obstructed labour in Eastern Uganda.

Variable	CRR 95% CI	P-value	ARR (95% CI)	P-value
**Age**				
Less than 3 years	1		1	
3 years and above	0.95(0.86–1.05)	0.317	0.95(0.86–1.04)	0.266
**Sex**				
Male	1		1	
Female	0.93(0.84–1.03)	0.144	0.91(0.82–1.01)	0.074
**Umbilical arterial lactate**				
< 4.8mmol/L	1		1	
4.8–10mmol/L	1.02(0.90–1.15)	0.766	1.06(0.95–1.18)	0.323
> 10 mmol/L	1.01(0.89–1.15)	0.866	1.054(0.93–1.20)	0.421
**Stunting**				
No	1		1	
Yes	1.03(0.92–1.14)	0.623	1.01(0.90–1.13)	0.904
**Blood pressure**				
Normal blood pressure < 90th percentile	1		1	
Elevated blood pressure ≥ 90th percentile	1.07(0.97–1.17)	0.168	1.09(0.99–1.19)	0.068
**Random blood sugar**				
RBS numerical	0.97(0.93–1.02)	0.251	0.98(0.93–1.03)	0.365
**Wealth Index**				
Upper income status	1		1	
Middle income status	1.01(0.89–1.13)	0.931	0.96(0.84–1.10)	0.535
Lower middle income status	0.99(0.90–1.10)	0.882	0.96(0.86–1.06)	0.419
**Exclusive breast feeding**				
Yes	1		1	
No	0.94(0.85–1.03)	0.166	0.95(0.86–1.06)	0.379
**Hospitalization with severe malaria in past one year**				
No	1		1	
Yes	0.89(0.77–1.03)	0.117	0.88(0.76–1.03)	0.126
**Food Diversity**				
No	1		1	
Yes	0.91(0.82–1.01)	0.079	0.95(0.85–1.06)	0.385

## Data Availability

The datasets used and/or analyzed during the current study are available from the corresponding author.

## Abbreviations

CKD: Chronic kidney disease
AKI: Acute kidney injury
MRRH: Mbale regional referral hospital
eGFR: Estimated glomerular filtration rate
